# Developing a Continuous Quality Improvement Assessment Using a Patient-Centered Approach in Optimizing Systemic Lupus Erythematosus Disease Control

**DOI:** 10.7759/cureus.1762

**Published:** 2017-10-09

**Authors:** Katelyn Mariko Updyke, Brittany Urso, Shazia Beg, James Solomon

**Affiliations:** 1 University of Central Florida College of Medicine; 2 Medical Student, University of Central Florida College of Medicine; 3 Rheumatology, University of Central Florida College of Medicine; 4 Dermatology, University of Central Florida College of Medicine

**Keywords:** systemic lupus erythematosus, social media, molecular mimicry, autoimmunity, antinuclear antibodies, autoantibodies

## Abstract

Systemic lupus erythematosus (SLE) is a multi-organ, autoimmune disease in which patients lose self-tolerance and develop immune complexes which deposit systemically causing multi-organ damage and inflammation. Patients often experience unpredictable flares of symptoms with poorly identified triggers. Literature suggests exogenous exposures may contribute to flares in symptoms. An online pilot survey was marketed globally through social media to self-reported SLE patients with the goal to identify specific subpopulations who are susceptible to disease state changes based on analyzed exogenous factors. The pilot survey was promoted for two weeks, 80 respondents fully completed the survey and were included in statistical analysis. Descriptive statistical analysis was performed on de-identified patient surveys and compared to previous literature studies reporting known or theorized triggers in the SLE disease state. The pilot survey identified similar exogenous triggers compared to previous literature, including antibiotics, increasing beef intake, and metal implants. The goal of the pilot survey is to utilize similar questions to develop a detailed internet-based patient interactive form that can be edited and time stamped as a method to promote continuous quality improvement assessments. The ultimate objective of the platform is to interact with SLE patients from across the globe longitudinally to optimize disease control and improve quality of care by allowing them to avoid harmful triggers.

## Introduction

Systemic lupus erythematosus (SLE) is an autoimmune disease that can affect multiple organ systems. SLE develops through a series of multiple steps, including loss of self-tolerance and the development of autoantibodies to self-deoxyribonucleic acid (DNA), known as anti-double-stranded-DNA (anti-dsDNA), occurring chronically for up to several years before symptom onset. This process generates anti-nuclear antibodies (ANA) involved in the formation of immune complexes that cause multi-organ damage. Symptoms of SLE disease exacerbation can include fatigue, fever, joint pain or swelling, and rash [1].

SLE symptom flares are associated with interactions between autoantibodies and circulating self-DNA. Multiple exogenous factors and exposures are thought to provoke SLE flares through several mechanisms. These mechanisms include molecular mimicry, wherein exogenous agents such as foods or chemicals contain cross-reacting antigenic epitopes and factors that alter DNA configuration (i.e., metal cations). SLE patients may be exposed to these exogenous triggers through ingestion, inhalation, injection, or direct skin or mucosal contact. The disease course in SLE is marked by unpredictable flares which have poorly identified triggers. It is currently hypothesized that certain exposures to ingestion of beef source, metals, medications, and more can contribute to the severity of SLE in patients [2]. It is still unclear which of these exposures affect which individuals, when, as well as whether or not these factors interact with each other or with other factors that have yet to be identified [3].

Even though self-nucleic acids are presumed to be the etiologic agent causing disease, the testing methods used to identify ANA, anti-dsDNA, and other lupus-related autoantibodies do not utilize healthy human DNA as reagents. Instead, the reagents used for these tests are usually DNA extracted from calf thymus, rabbit, parasites (i.e., Crithidia luciliae) and cancerous human cells [i.e., Helacyton gartleri derived, also known as, HeLa or HEp-2 cells) [4-6]. Reactions to these reagents are correlated with anti-self DNA; however, these testing methods also demonstrated that there is a reaction to the DNA from the source reagent. Despite a generally accepted concept that DNA segments from food sources do not enter circulation, studies show that DNA recognizable to its food source can be found in circulation in humans within one hour of ingestion [7]. Animal models confirm this as well as find food source DNA incorporated into internal organ nuclear DNA [8]. A study conducted previously showed that the gastrointestinal tract of mammals is the main portal of entry for foreign DNA and proteins. It was found that, for up to 18 hours after food ingestion, fragments of foreign DNA could be visualized by fluorescence in situ hybridization in cecal epithelia [9]. Some literature has suggested that alterations in diet may contribute to autoimmunity [10]. However, definitive evidence is still lacking in this area of study. At the Translational Science 2014 Annual Meeting in Washington D.C., Solomon et al. presented "Identifying the Role of Molecular Mimicry in SLE Immune Complex Disease" and reported that some SLE patients who follow a vegan diet have improved or obtained complete control of their symptoms. This data suggests that there are subpopulations of SLE patients whose disease state may be influenced by their diet through the cross-reaction of food source DNA with self. This molecular mimicry may cause disease through the formation of immune complexes in SLE patients. A well-known example of molecular mimicry is seen in the autoimmune model of rheumatic heart disease (RHD). In RHD, patients may relapse with exposure to streptococci carrying antigens which cross-react with human cardiolipin [11]. Thus, the target antigen in SLE patients with active immune complexes may not need to be self-antigen [12-13].

Several different external and environmental influences have been explored in regards to their contribution to SLE development and symptoms. Some commonly prescribed drugs which are considered safe for most patients may have the potential to worsen existing immune complex disease or produce a drug-induced lupus syndrome. Prior case studies report SLE exacerbations in patients treated with Griseofulvin, an anti-fungal medication, but the mechanism is unknown. In one SLE patient, the provocation of SLE symptoms was so severe that it proved to be fatal [14]. Other commonly prescribed and relatively safe drugs, such as beta-lactam antibiotics are capable of activating cells of the immune system resulting in the preferential formation of serum antigens [15].

Immune cell behavior can be influenced by the presence of metal-complexing agents [16]. In fact, lead is capable of exacerbating SLE symptoms in lupus-prone mice. The degree of exacerbations is determined by the genetic differences between these mice [17]. Furthermore, an SLE patient developed a malar rash and was diagnosed with a delayed-type hypersensitivity reaction to nickel and possibly cobalt dichloride after wearing metal framed eyeglasses. The patient’s malar rash mimicked the rash caused by acute cutaneous lupus erythematosus and completely resolved with her avoidance of contact with her eyeglass frames [18]. This prompted us to explore SLE patient sensitivity to other types of metal and whether these metals influence their symptoms. 

Currently, almost 50% of the world's population uses the internet with over 1.5 billion individuals using Facebook every day. Of the 2.5 million monthly posts related to skin disease, lupus accounts for about 500,000 social media posts per month. Social media was chosen to gain insights from motivated patients who are likely to be playing an important role in establishing patient-oriented outcomes.

The purpose of this study was to construct a pilot survey that will be utilized to develop a Patient Interactive Form (PIF). The pilot survey was extensive in its questioning, revealing that motivated patients will complete the study despite "survey burnout". The pilot study identified similar triggers compared to some reported in the literature. We propose that molecular mimicry plays a role in a subset of SLE patients, and we hope to identify triggers that may aggravate their symptoms and report them back to patients. Ultimately, we hope to improve their quality of life through our new PIF platform by helping them to make the lifestyle changes necessary that may help to control their symptoms.

## Materials and methods

Development of the pilot survey

Extensive literature review of SLE revealed multiple exogenous factors that may be involved in SLE relapses and remissions. An online pilot survey was designed based on the reviewed literature of known SLE triggers and can meet several significant components of high-value health care delivery, such as considering a patient’s medical condition, their geographic region, and measuring costs and outcomes for each patient [19]. The pilot survey was released in English and is available through a SurveyMonkey® link (https://www.surveymonkey.com/r/6HMB993). 

Participant recruitment

Participation consent was received from all participants prior to questionnaire initiation. The pilot survey was internationally promoted to adults, aged 18 or older, who self-identified as having been diagnosed with systemic lupus erythematosus. Participants were recruited via various social media platforms through the internet (Facebook, Instagram, Twitter, etc.) and provided a link, which directed them to the study. The International Federation of Dermatology Clinical Trials promoted the survey through its membership. Participant information was collected through SurveyMonkey® and was de-identified per the Health Insurance Portability and Accountability Act of 1996 (HIPAA).

Data analysis

The pilot survey data was de-identified and analyzed using IBM Statistical Package for Social Sciences (SPSS) software, version 23.0, (IBM Corp., Armonk, NY) in order to identify relationships between changes in disease state and external factors in SLE patients. Categorical (gender, ethnicity, residence, antibiotic use, materials used in surgical and dental procedures), dichotomous (surgical history, prior dental procedure, exacerbation in symptoms), and ordinal (degree of symptom severity, normal amount of dietary beef intake, changes in beef intake within 30 days) variables were studied. Relationships were determined between two categorical variables (i.e., the material used in a surgical procedure and exacerbation of symptoms) using cross-tabulations. Categorical variables are represented as frequencies and percentages. Thus, there are no testing parameters given the descriptive nature of this research study.

## Results

The questionnaire consisted of 148 questions over 63 pages and the time estimated for completion was 90 minutes. This questionnaire captured baseline data collection on overall health, medications, surgeries as well as lifestyle, diet, and environmental exposures. Patients were also asked a series of questions regarding their disease state within the past 30 days which included the worst symptoms they were experiencing, the severity of symptoms, and the frequency of SLE flares in a week. Additionally, patients were asked whether they had prior exposures to specific external factors and whether those external factors contributed to flares in their SLE symptoms. 

After two weeks of promoting the survey, there were 178 respondents, in which 80 participants completed the survey to its end and were included in statistical analysis. There was one participant who declined consent and was disqualified from the study. Patients of all genders, race, or ethnicity could participate in the study and study participation was strictly voluntary. Socioeconomic status may limit some populations of patients from accessing the study since this study requires computer and internet access to complete. Patients could skip any questions they did not feel comfortable answering and could discontinue the survey at any point. The SurveyMonkey® platform requires participants to complete the survey in one session. These SLE patients reported exacerbations (flares) related to exogenous exposures from molecular mimicry cross-reactive epitopes (i.e. dietary beef). As theorized, they also reported exacerbation associated with metals (i.e., nickel, gold, etc.) through dental procedures and surgical implants.

Only women completed the survey; however, one patient chose not to disclose their gender (Table 1). Most patients who responded to the survey were Caucasian or European and resided in the United Kingdom (Table 1). Patients could check all ethnicities that applied to them. There was one respondent who did not report where they reside.

**Table 1:** Demographics of surveyed self-reported SLE patients. Patients were able to select more than one ethnicity if applicable. ^1^One patient did not self-report their sex. ^2^Patients who reported themselves as "White" were grouped in Caucasian. Those who reported themselves as English, British, Scottish, Irish, Welsh, White British, Black British or Romanian were listed as European. ^3^One patient did not report where they reside.

Sex (n =79)^1^		Residence (n=79)^3^		Residence (n=79)^3^	
Female	79	Outside of the United States		United States	
Male	0	Australia	2	California	3
Ethnicity/Race (n=80)		Austria	1	Illinois	1
African American	1	Ireland	2	New York	1
Latino/Hispanic	1	United Kingdom	65	Ohio	1
Caucasian	39			Texas	1
Australian/New Zealander	1			Washington	1
Asian	2			West Virginia	1
European^2^	38				
Middle Eastern	1				

Patients were surveyed on their regular dietary beef intake (n=78). The majority of respondents eat beef on a weekly basis (46%), while a minority of patients eat beef daily (4%). However, the rest of participants responded that they eat beef monthly (19%), rarely eat beef (15%), or never eat beef (15%). Respondents indicated whether their dietary intake of beef had changed within 30 days prior to them completing the survey (n=63). The majority of patients indicated that their intake has been unchanged in the past 30 days (79%); however, there were nine respondents who decreased their beef intake (14%) and four respondents who increased their beef intake (6%) (Table 2).

**Table 2:** Dietary beef intake habits. Data are presented in frequency and percentage [i.e., n (%)]. ^1^Two did not respond. ^2^Seventeen did not respond.

Regular dietary intake of beef (n=78)^1^				
Daily	Weekly	Monthly	Rarely	Never
3 (4)	36 (46)	15 (19)	12 (15)	12 (15)
Changes in beef ingestion in the last 30 days (n=63)^2^				
Increase	Unchanged	Decrease		
4 (6)	50 (79)	9 (14)		

Relationships were determined between change in dietary intake of beef and severity of SLE symptoms patients were experiencing in the past 30 days. Patients were asked how they felt within 30 days of completing the survey. They could respond as “very well, with no to minimal symptoms,” “good with minimal to mild symptoms,” “average with moderate symptoms,” “poor, with moderate to severe symptoms,” or “very poor, with consistent severe symptoms.” All respondents who indicated that they increased their dietary beef intake also noted that they were experiencing severe consistent SLE symptoms in the past 30 days (100%). Strength of symptoms among those who did not change their beef intake were scattered with 6% of respondents having no to minimal symptoms, 22% of patients with minimal to mild symptoms, 34% of patients with moderate symptoms, 28% of patients experiencing moderate to severe symptoms, and 10% of patients experiencing severe symptoms. Lastly, those who decreased their dietary intake of beef experienced a moderate degree of SLE symptoms in the past 30 days (33%), moderate to severe symptoms (33%), or severe symptoms (33%) (Table 3).

**Table 3:** Relationship between change in beef intake and strength of SLE symptoms. Seventeen did not respond and were not included (n=63). The strength of symptoms is shown in the far-left column and the change in beef intake is shown on the top row. Four patients increased their beef intake, fifty did not change their beef intake and nine decreased their beef intake within the past 30 days of taking the survey. Data is presented in frequency and percentage [i.e. n (%)].

	Increase (n=4)	Unchanged (n=50)	Decrease (n=9)
None to minimal	0 (0)	3 (6)	0 (0)
Minimal to mild	0 (0)	11 (22)	0 (0)
Moderate	0 (0)	17 (34)	3 (33)
Moderate to severe	0 (0)	14 (28)	3 (33)
Severe	4 (100)	5 (10)	3 (33)

Respondents who have previously been prescribed antibiotics were asked whether they experienced an exacerbation in their SLE symptoms with its use. The majority of exacerbations experienced were with cefuroxime (71%), trimethoprim-sulfamethoxazole (60%), and Nitrofurantoin (44%). However, there were also adverse responses in SLE patients with the use of minocycline (33%), penicillin (31%), and tetracycline (29%) (Table 4). There were 33 respondents who indicated being prescribed at least one of the antibiotics listed, 19 participants were prescribed two antibiotics listed, and four participants reported being prescribed three or more of the antibiotics listed. However, there were 24 respondents who indicated they had not been prescribed any of the antibiotics listed. Additionally, surveyed participants who received metal prosthetic implants also experienced exacerbations in their SLE symptoms (29%) within six months of its placement, while those who had plastic prosthetic implants did not. Dental procedures using foreign materials were also studied in SLE patients. Patients who received gold crowns did not experience any negative change in their disease state within six months of the procedure. However, patients reported negative change in their SLE with silver crowns (31%), porcelain crowns (29%), other metal caps (29%), and with bridges or false teeth (23%) (Table 4). There were 28 respondents who indicated having a procedure with only one of these materials, 11 participants had procedures with two of the materials listed, and two participants reported having a procedure using three or more of the materials listed in the question. Furthermore, a population of patients who had acrylic or white plastic dental fillings experienced exacerbations in their symptoms (24%) within six months of the procedure, as well as those who received fillings using gold (50%), silver (20%) or other metals (28%) (Table 4). There were 39 participants who reported having only one of these materials used in their dental fillings, 16 patients who reported having dental fillings with two of these materials, and three patients who had three or more of the materials used in their dental fillings.

**Table 4:** Potential triggers of SLE flares. Data are presented in frequency and percentage [i.e., n (%)]. TMP-SMX is trimethoprim-sulfamethoxazole. ^1^Participants could select more than one response. ^2^One did not respond but was prescribed both Cefuroxime and Tetracycline previously. ^3^Twenty-four did not respond. ^4^One did not respond and received a plastic prosthetic. ^4^Three did not respond.

Antibiotics prescribed in the past (n=80)^1^		Exacerbations (n=79)^2^	
Cefuroxime	7	5 (71)	
Penicillin	49	15 (31)	
TMP-SMX	5	3 (60)	
Tetracycline	17	5 (29)	
Nitrofurantoin	9	4 (44)	
Minocycline	3	1 (33)	
Surgical Prosthetic Placement (n=10)^3^		Exacerbations (n=9)^1^	
Plastic	3	0 (0)	
Metal	7	2 (29)	
Dental Procedure (n=65)^1,3^		Exacerbations (n=65)	Unknown (n=65)
Silver Crown	13	4 (31)	5 (38)
Gold Crown	4	0 (0)	1 (25)
Porcelain Crown	21	6 (29)	8 (38)
Other metal caps	7	2 (29)	2 (29)
Bridges or false teeth	13	3 (23)	6 (46)
Dental Fillings (n=61)^1,4^		Exacerbations (n=61)	Unknown (n=61)
Acrylic or white plastic	33	8 (24)	14 (42)
Gold	2	1 (50)	1 (50)
Silver	20	4 (20)	10 (50)
Other metal	25	7 (28)	11 (44)

## Discussion

Based on the demographics of the data analysis, there were only self-reported females who completed the survey. It is well known that autoimmune diseases are much more frequent in women, in which female hormones may interact with the immune response and play a role in its pathogenesis [20]. The greatest population of respondents resided in the United Kingdom, and most patients reported their ethnicity as Caucasian. The second greatest patient population self-reported as European. There were very few respondents who resided in the United States or other countries other than the United Kingdom. This may be because our most supportive organizations who promoted the survey, after contacting them via social media platforms, were in the United Kingdom (i.e. Lupus UK, Cochrane Group). Additionally, there were few self-reported minorities who participated in the survey as well. These vast differences in patient population which responded to the survey may have been influenced by participant recruitment methods. Patients were solely recruited via social media which required patients to have internet access and accounts with various social media websites (e.g., Facebook, Twitter, etc.). The subpopulations of people without internet access or social media accounts may include individuals who are underprivileged, spend little time online, or naïve to social media websites. 

We recognize that SLE patients make autoantibodies to their own tissues, but also understand that there have been previous studies that propose nonhuman DNA can contribute to SLE. The pilot survey included questions regarding other known, non-self DNA sources that could induce lupus exacerbations or the onset of SLE itself. One of these sources is ingested animal protein. The survey captured the dietary baseline amounts of beef that the responding SLE patients ate on a regular basis. The surveyed patients were also asked whether they had changed the amount of consumption of beef in the last 30 days. Every patient who reported increasing their dietary beef intake also indicated that they were having consistently severe SLE symptoms within 30 days of taking the survey. There have been animal feeding studies done in the past which have demonstrated that a minor amount of fragmented dietary DNA may resist digestion; however, the fate of this undigested DNA is still not completely understood [21]. It appears that excessive caloric intake, excess protein, and high-fat content (especially omega-6 polyunsaturated fats) can be aggravating dietary substances in lupus patients. However, there have been no large-scale studies completed on lupus patients that can substantiate any benefits from dietary interventions [22]. Furthermore, it has been previously found that antibodies to two commonly ingested proteins, bovine gamma globulin and bovine sera albumin, have been found in the sera of SLE patients. Moreover, this demonstrates the potential role, as well as the need for careful evaluation of the wide variety of different foods which may behave as antigens in immune complex diseases [23]. This provides us with the unique opportunity to interact with a greater population of SLE patients worldwide, over extended time periods, to capture patterns in disease flares compared to different dietary habits.

We explored the relationship between antibiotic use and SLE disease flare. The greatest number of patients we surveyed experienced adverse reactions with Cefuroxime and trimethoprim-sulfamethoxazole. Cefuroxime is within the beta-lactam family and trimethoprim-sulfamethoxazole contains a sulfonamide. The global pilot survey captured similar results as a prior study that surveyed a subgroup of patients only within the John Hopkins Lupus Cohort. The John Hopkins study found that the highest percentages of antibiotic allergies were among SLE patients exposed to Penicillin/Cephalosporin and sulfonamides, with worsening of symptoms associated mostly with sulfonamides [24].

Similarly, we investigated the ability of different metals’ effect on the SLE disease state. It has been found that metal-complexing agents can alter DNA structure and modulate immune cells [16]. Thus, it was determined that there is a relationship between metals used in dental restorative procedures and changes in the SLE disease state. Metals used in dental restorations often contain nickel, gold, and mercury which chronically expose people to metal ions through corrosion and leads to metal-induced delayed hypersensitivity reactions [25]. In the survey, we identified several SLE patients who adversely reacted to metal crowns and fillings. Stejskal, et al. studied delayed-type hypersensitivity reactions to metals in patients with various connective tissue diseases. This study surveyed 38 patients who were diagnosed with connective tissue disease, in which nine patients had SLE. The most frequent allergens reported were nickel, gold, palladium, and mercury, which were mainly found in dental restorations [25]. The pilot survey was able to capture similar experiences in worsening of symptoms with dental procedures as reported by the 80 participating patients across the globe. None of the surveyed participants experienced worsening of their SLE with gold crowns, but one of two patients had adverse events in their SLE with gold dental fillings. Furthermore, some participants had a worsening of SLE symptoms with porcelain crowns. Porcelain can contain titanium and aluminum, which may be a source of metal-induced hypersensitivity in SLE patients [26]. 

In previous literature, SLE patients have experienced malar rashes from wearing metal-framed eyeglasses due to a delayed type IV hypersensitivity reaction [27]. Thus, we explored whether surgical intervention in patients that include placement of foreign materials (i.e., metal or plastic prosthetics, metal dental fillings or crowns, etc.) showed increased frequencies of symptom flares in SLE patients. The pilot survey results identified a subset of patients who had a metal prosthetic placement surgery and experienced an exacerbation of their symptoms within six months of the procedure, whereas no participants reported having an exacerbation with plastic implants. Loyo, et al. described an isolated case study of a 23-year-old woman who had a nickel-titanium chin implant and developed antinuclear antibodies one year after surgery. Additionally, she developed symptoms of patients diagnosed with SLE and antiphospholipid syndrome. Her systemic symptoms resolved completely after she removed her metal chin implant [28]. These findings suggest the importance of the roles that metals may play in the pathogenesis of SLE and the need for further careful evaluation of their ability to induce flares in lupus patients. None of the surveyed SLE patients had an exacerbation of their symptoms with a plastic implant placement procedure.

SLE is an immune-complex disorder which can improve or exacerbate through several scenarios that make the ability to determine cause-effect more difficult, but with enough data, it is not impossible. Thus, an SLE patient in exacerbation may have immune complexes which can disassociate from antigen excess or antibody excess. Moreover, reduced exposure to the antigen leads to improvement through antibody excess. On the other hand, an increased exposure to the antigen may also lead to improvement through antigen excess. Once additional antibodies are produced, immune complexes re-form and exacerbation of symptoms can occur again. From a different perspective, patients may enter remission due to antigen or antibody excess. Therefore, additional antigen may cause disease exacerbation, whereas reduced antigen levels may maintain remission for a patient in remission, secondary to antibody excess. For patients in remission due to antigen excess, decreased exposure to the antigen may lead to exacerbation while increased antigen leads to continued remission. Our group is developing the appropriate questions to address these issues. 

This pilot survey initiated the use of online social media platforms on a global scale to interact with SLE patients. We captured the responses of several SLE patients from varying residences and compared their experiences with those recorded in previous literature. It is important to recognize that molecular mimicry may be playing a role in disease exacerbation in a subset of SLE patients.

The pilot survey identified similar exogenous triggers compared to previous literature, which includes metals used in surgical and dental procedures, antibiotic use, and increased ingestion of dietary beef. The goal of the study is to use the information gained from this study to develop a highly detailed internet-based patient interactive form (PIF) which patients are able to log in and out at their own convenience. Additionally, common belief is that most participants experience “survey burnout"; however, we were able to achieve good participation with a lengthy questionnaire. We propose that patients with chronic illnesses with symptoms that are difficult to control are more motivated to participate in studies with a greater goal of improving their quality of life [29]. 

The authors have developed a hardware-software platform to allow participants to login and logoff, at will, without revealing HIPAA protected data. The information gained from this pilot survey will be used to help us develop an extensive PIF that will track patients longitudinally over a period of years, continuously capturing dynamic changes in their SLE symptoms based on their lifestyles. Questions on the PIF will be tailored to evaluate SLE patient responses to known exogenous triggers, as well as explore and evaluate other external factors that may contribute to the pathogenesis of SLE. Data captured will be analyzed using complex adaptive systems (CAS) methodology, which finds arrays of factors that are associated with similar outcomes in patients’ disease status. We hope to identify complex patterns of worsening in SLE symptoms based on triggers they encounter on a daily basis. We plan to report these results back to patients, allowing them to make changes in their habits that will better control their symptoms. Ideally, as patients continue to use our PIF, their symptoms should improve, ultimately lowering the cost of care in this subset of patients.

## Conclusions

Systemic lupus erythematosus is a relatively common, yet complex, autoimmune disease that has symptoms that can be difficult to control. We propose that molecular mimicry plays a role in a subset of lupus patients that can contribute to disease flares and lower quality of life. We constructed a pilot survey that was promoted to lupus patients via social media and we identified: 1) a subset of SLE patients using social media are motivated to participate in a lengthy study, 2) a subset of SLE patients may have worsening of symptoms with certain triggers (i.e., antibiotics, metal implants, ingestion of beef), and 3) the data needed to help develop a new patient interactive form that will be used to track lupus patients longitudinally. Our new patient interactive form will be utilized to help SLE patients identify triggers they encounter on a daily basis that may be helping or worsening their disease state so that they can make the necessary lifestyle changes to improve their health and take control of their disease.
